# The association between provider characteristics and post-catheterization interventions

**DOI:** 10.1371/journal.pone.0266544

**Published:** 2022-04-01

**Authors:** Adam C. Powell, Jason P. Goldstein, James W. Long, Jeffrey D. Simmons, Anthony DeFrance

**Affiliations:** 1 HealthHelp, Houston, TX, United States of America; 2 Humana Inc., Louisville, KY, United States of America; Medizinische Universitat Graz, AUSTRIA

## Abstract

**Objectives:**

To examine whether the demographics of providers’ prior year patient cohorts, providers’ historic degree of catheter-based fractional flow reserve (FFR) utilization, and other provider characteristics were associated with post-catheterization performance of percutaneous coronary intervention (PCI) or coronary artery bypass grafting (CABG).

**Study design:**

A retrospective, observational analysis of outpatient claims data was performed.

**Methods:**

All 2018 outpatient catheterization claims from a national organization offering commercial and Medicare Advantage health plans were examined. Claims were excluded if the patient had a prior catheterization in 2018, had any indications of CABG or valvular heart disease in the prior year of claims, or if the provider had ≤10 catheterization claims in 2017. Downstream PCI and CABG were determined by examining claims 0–30 days post-catheterization. Using multivariate mixed effects logistic regression with provider identity random effects, the association between post-catheterization procedures and provider characteristics was assessed, controlling for patient characteristics.

**Results:**

The sample consisted of 31,920 catheterization claims pertaining to procedures performed by 964 providers. Among the catheterization claims, 8,554 (26.8%) were followed by PCI and 1,779 (5.6%) were followed by CABG. Catheterizations performed by providers with older prior year patient cohorts were associated with higher adjusted odds of PCI (1.78; CI: 1.26–2.53), even after controlling for patient age. Catheterizations performed by providers with greater historic use of FFR had significantly higher adjusted odds of being followed by PCI (1.73; CI: 1.26–2.37).

**Conclusion:**

Provider characteristics may impact whether patients receive a procedure post-catheterization. Further research is needed to characterize this relationship.

## Introduction

Managed care organizations, patients, and referring providers all have a vested interest in understanding how modifiable factors impact patient outcomes. One readily modifiable and observable factor is the provider performing the care. Provider characteristics, such as practice patterns in the prior year and the demographics of providers’ cohorts of patients in the prior year, are observable features that can be used to guide decisions regarding where patients should receive catheterization.

Although factors external to the condition of the patient being treated should not influence care, empirical research suggests that this is not the case. While normatively, the characteristics of the other patients that a provider treated in the past year should not influence the outcomes that a particular patient experiences after catheterization, descriptively, they may influence outcomes, as providers develop practice patterns in response to the characteristics of the other patients that they have previously treated. Patient panel characteristics have been found to be significantly associated with performance on a composite measure of care quality in the context of primary care [1]. Likewise, while normatively, practice environment characteristics such as urbanicity should not influence care, descriptively, urbanicity has been shown to have a significant association with screening and monitoring quality measure outcomes, as well as avoidable utilization [2].

One provider characteristic with the potential to impact post-catheterization outcomes is the provider’s propensity to perform catheter-based fractional flow reserve (FFR). Several sets of clinical guidelines had provided a moderate level of endorsement for the use of FFR measurement, but FFR was not yet elevated to being part of the American standard of care at the time the data were collected [3–5]. It was only in 2021 that the ACC/AHA/SCAI Guideline for Coronary Artery Revascularization replaced a prior guideline, and introduced a strong (Class 1) recommendation for the use of FFR based upon high-quality (Level A) evidence [6]. Therefore, there is likely both provider-level variation in the use of FFR, and among providers who perform it, variation in the extent to which they do so. While there are a number of different alternatives to FFR that have been developed, this study focuses on FFR utilization as FFR is the gold standard for assessing intermediate coronary stenosis, and was the first functional severity assessment developed [7, 8].

Although coronary angiograms enable physicians to visually approximate the degree of stenosis within the heart, they provide only a two-dimensional, anatomical view that is susceptible to inter-observer variability [9]. To supplement this information, physicians can measure the pressure difference across stenoses by performing FFR during catheterization procedures. This additional information can empower physicians to have a more complete understanding of the hemodynamic importance of a stenosis when deciding whether to perform a percutaneous coronary intervention (PCI) or coronary artery bypass grafting (CABG) surgery.

There is currently evidence that suggests that FFR is useful in guiding the use of PCI, but not CABG [10, 11]. Deferring PCI based upon FFR findings (FFR ≥ 0.75) has been shown to result in five-year event-free survival that does not differ significantly from what is seen in similar patients undergoing immediate PCI [12]. The benefits of deferring PCI based upon FFR findings have furthermore been shown to be observed when there is fifteen year follow-up [13]. When providers perform FFR, in some cases, they will identify situations in which patients do not need to undergo PCI, and may likewise identify lesions that anatomically appear to be non-flow limiting, but in fact require PCI. Identifying patients in need of PCI is important for patient health, as reversing myocardial ischemia through revascularization rather than medical therapy has been shown to confer a greater survival benefit in patients with moderate to large amounts of inducible ischemia [14]. Furthermore, a meta-analysis of individual patient data from three randomized trials of FFR-guided PCI versus medical therapy for patients with stable coronary lesions concluded that FFR-guided PCI reduced the composite risk of death or myocardial infarction, compared with medical therapy [15].

Although associations between provider characteristics and clinical outcomes have been established in other contexts, they have yet to be explored in the context of cardiac catheterization. As performing FFR during catheterization had not become a part of the American standard of care during the period of observation, there may be substantial variation in the extent to which providers performed it, which in turn may impact outcomes. Likewise, as the demographics of prior patients have been shown to influence care in other contexts, they may be an additional driver of post-catheterization outcomes. The purpose of this study was to assess whether observable provider characteristics were associated with post-catheterization performance of percutaneous coronary intervention (PCI) or coronary artery bypass grafting (CABG) surgery. If significant associations are found, further research may be needed to determine which observable provider characteristics should be considered when selecting a provider for cardiac catheterization.

## Methods and materials

### Ethics statement

This study has received an Institutional Review Board exemption from Advarra IRB (Pro00041605). Consent was not obtained, as this was a retrospective, observational analysis, and patients were de-identified. Re-identifying them to contact them would pose a risk to patient privacy. The study was conducted in accordance with the principles of the Declaration of Helsinki.

### Data source and sample population

This retrospective, observational study consisted of a patient-level claims analysis that was clustered by provider, as many patients were treated by the same providers. Claims data covering care delivered from January 1^st^, 2017 to January 31^st^, 2019 were extracted from the database of a national healthcare organization offering commercial and Medicare Advantage health plans. Health plan enrollment data for this period was also extracted. Outpatient catheterization claims from 2018, pertaining to patients aged 18 to 89 years, satisfied the inclusion criteria for the study. Claims were excluded if the patient to which they pertained was not continuously enrolled in their health plan from twelve months prior to catheterization to a month post-catheterization, if the patient had a previous claim for cardiac catheterization in calendar year 2018, if the patient had claims indicating presence of valvular heart disease (ICD-10 codes: I34.*, I35.*, I36.*, and I37.*) in the prior year, if the patient had claims for CABG (see S1 Appendix) or indicating presence of a CABG (ICD-10 code Z95.1) in the prior year, or if the provider that performed the catheterization had performed ten or fewer cardiac catheterization claims in calendar year 2017 on patients with health plans from the national organization. (This requirement also ensured that only claims from providers performing catheterization in both 2017 and 2018 were included in the analysis.) To avoid double-counting, claims were attributed to providers based upon the professional component, and if none was available, the global component. Lists of the Current Procedural Terminology (CPT) codes used in the analysis are provided in S1 Appendix.

Several control variables were constructed by mapping the data to other data available from public sources. Median income in patients’ ZIP codes was determined using the American Community Survey’s 2013–2017 5-year estimates, reporting income in 2017 inflation-adjusted dollars [16]. The prevalence of obesity in each state was determined using 2018 data from the Behavioral Risk Factor Surveillance Survey released by the Centers for Disease Control and Prevention [17]. A ZIP Code mapping table developed by the Centers for Medicare & Medicaid Services was used to determine whether patients’ ZIP Codes were urban or rural [18].

### Measurement

#### Outcomes

The outcomes examined in the study were whether the patient had a PCI or a CABG performed by any provider within 0 to 30 days from the date of the claim for the catheterization.

#### Provider characteristics

Provider characteristics served as the independent variables in the analysis, while patient characteristics served as the control variables. Providers were identified using tax identification numbers, and consisted of one or more physicians billing under a common tax identification number. The patient cohort-related, provider-level covariates corresponded to the demographics of the patients receiving catheterization from the provider in the prior year, 2017. The provider-level independent variables considered were the ratio of the provider’s prior year FFR visits to catheterization visits, if the site of service was an on-campus outpatient hospital setting, ambulatory surgical center, or office, the mean age of the provider’s patients, the percentage of the provider’s patients that were male, and the proportion of the provider’s patients from low income (below $40,000 median income), high income (above $80,000 median income), and urban settings.

#### Patient characteristics

The patient-level covariates considered were age, sex, number of prior catheterizations in the prior 12 months, whether the patient lived in a ZIP code with a median income below $40,000 or above $80,000 per year, the urbanicity of the patient’s ZIP code, and the obesity rate of the patient’s home state. Variables not falling between 0–1 inclusive were rescaled to fall within that range. Provider identity random effects variables were used to account for the fact that many providers were responsible for multiple claims.

### Analysis

#### Univariate analysis

Providers were divided into three terciles based on the tercile of their ratio of the number of visits in which they performed a catheterization-based FFR in 2017 to the number of visits in which they performed a catheterization in 2017. FFR computed tomography was outside the scope of this analysis, as it would likely be performed by a different provider than catheterization-based FFR. Descriptive statistics for the overall population, as well as for patients allocated to each tercile, were computed. For each of the variables examined, comparisons were made to determine if there were differences between terciles. Values for variables with binary outcomes (e.g., whether the patient lived in an urban location) were compared with Chi-Square tests, while values for variables with continuous outcomes (e.g., the patient’s age) were compared with one-way analysis of variance (ANOVA).

The percentage of 2018 catheterization claims followed by PCI and followed by CABG was determined for each tercile. Chi-Square tests were used to assess whether a significant association was present between a provider’s ratio of visits with an FFR claim in 2017 to visits with a catheterization claim in 2017, and the likelihood that a catheterization performed by the provider would be followed by PCI or CABG in 2018.

#### Multivariate analysis

Using multivariate mixed effects logistic regression with provider identity random effects, the association between post-catheterization outcomes (PCI, CABG) and provider characteristics was assessed, controlling for patient characteristics. Provider identity random effects were incorporated into the model to account for the fact that all the claims from each provider shared the same provider characteristics. Findings from the analysis were reported as adjusted odds ratios with 95% confidence intervals (CI), and a threshold of *P*<0.05 was used to determine significance.

## Results

**Fig 1:**
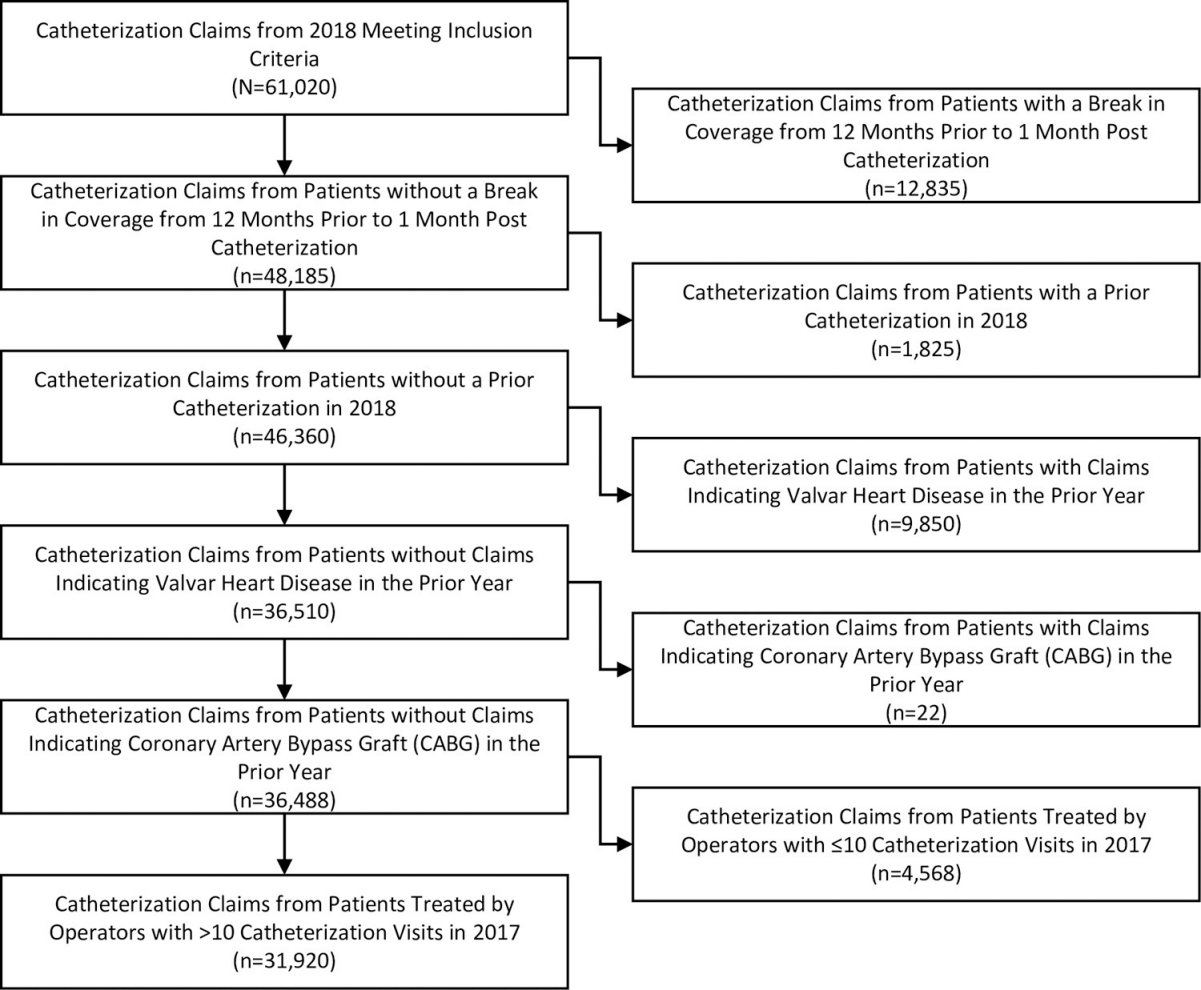
Sample selection diagram.

The sample consisted of 31,920 catheterization claims (Fig 1); 8,554 (26.8%) were followed by PCI and 1,779 (5.6%) were followed by CABG. The catheterization claims pertained to 964 different healthcare providers. When catheterization claims were divided according to whether the provider that performed the catheterization was in the lowest, middle, or highest tercile of FFR utilization, 8,648 claims came from providers in the lowest tercile, 13,705 came from providers in the middle tercile, and 9,567 came from providers in the highest tercile.

Descriptive statistics, grouped by the provider’s FFR / catheterization ratio tercile, are presented in Table 1. Patients receiving catheterization from providers in the tercile with the lowest propensity to perform FFR were treated by a provider with a mean FFR / catheterization ratio of 0.02. This mean was 0.11 and 0.26 for the middle and highest terciles respectively. The three groups significantly differed on all factors examined, except patient age and the number of catheterizations received in the prior 12 months.

**Table 1:** Descriptive statistics by provider’s FFR / catheterization ratio tercile.

	All (N = 31,920)	Lowest Tercile (n = 8,648)	Middle Tercile (n = 13,705)	Highest Tercile (n = 9,567)	*P* Value
***Provider Characteristics***					
Cohort FFR / Catheterization Visit Ratio in Prior Year, Mean ± SD	0.13 ± 0.11	0.02 ± 0.02	0.11 ± 0.03	0.26 ± 0.11	< .01
Place of Service: On-Campus Outpatient Hospital, n (%)	31,010 (97.15)	8,243 (95.32)	13,286 (96.94)	9,481 (99.10)	< .01
Place of Service: Ambulatory Surgical Center, n (%)	63 (0.20)	42 (0.49)	<10 (<0.07)	17 (0.18)	
Cohort Mean Age in Prior Year, Mean ± SD	70.28 ± 2.17	70.28 ± 2.22	70.32 ± 1.95	70.22 ± 2.4	< .01
Cohort % Male in Prior Year, Mean ± SD	0.57 ± 0.08	0.56 ± 0.09	0.57 ± 0.07	0.58 ± 0.08	< .01
Cohort Local Median Income: % <$40,000 in Prior Year, Mean ± SD	0.29 ± 0.19	0.32 ± 0.20	0.27 ± 0.16	0.27 ± 0.21	< .01
Cohort Local Median Income: % >$80,000 in Prior Year, Mean ± SD	0.06 ± 0.10	0.05 ± 0.09	0.06 ± 0.09	0.08 ± 0.11	< .01
Cohort % Urban in Prior Year, Mean ± SD	0.74 ± 0.25	0.71 ± 0.28	0.75 ± 0.22	0.75 ± 0.27	< .01
***Patient Characteristics***					
Age, Mean ± SD	70.73 ± 8.51	70.83 ± 8.39	70.66 ± 8.58	70.75 ± 8.51	0.37
Male, n (%)	18,614 (58.3)	4,925 (56.9)	8,044 (58.7)	5,645 (59.0)	<0.01
Total Catheterizations in the Prior 12 Months, Mean ± SD	0.04 ± 0.21	0.05 ± 0.22	0.04 ± 0.21	0.04 ± 0.20	0.92
Local Median Income: <$40,000, n (%)	9,130 (28.60)	2,872 (33.21)	3,654 (26.66)	2,604 (27.22)	< .01
Local Median Income: >$80,000, n (%)	2,009 (6.29)	429 (4,96)	868 (6.33)	712 (7.44)	
Urban, n (%)	23,637 (74.05)	6,127 (70.85)	10,307 (75.21)	7,203 (75.29)	< .01
State Obesity Rate, Mean ± SD	0.34 ± 0.03	0.34 ± 0.03	0.34 ± 0.03	0.34 ± 0.03	< .01

Abbreviations: FFR: Fractional Flow Reserve; SD: Standard Deviation.

Note: Counts suppressed when less than 10.

**Fig 2:**
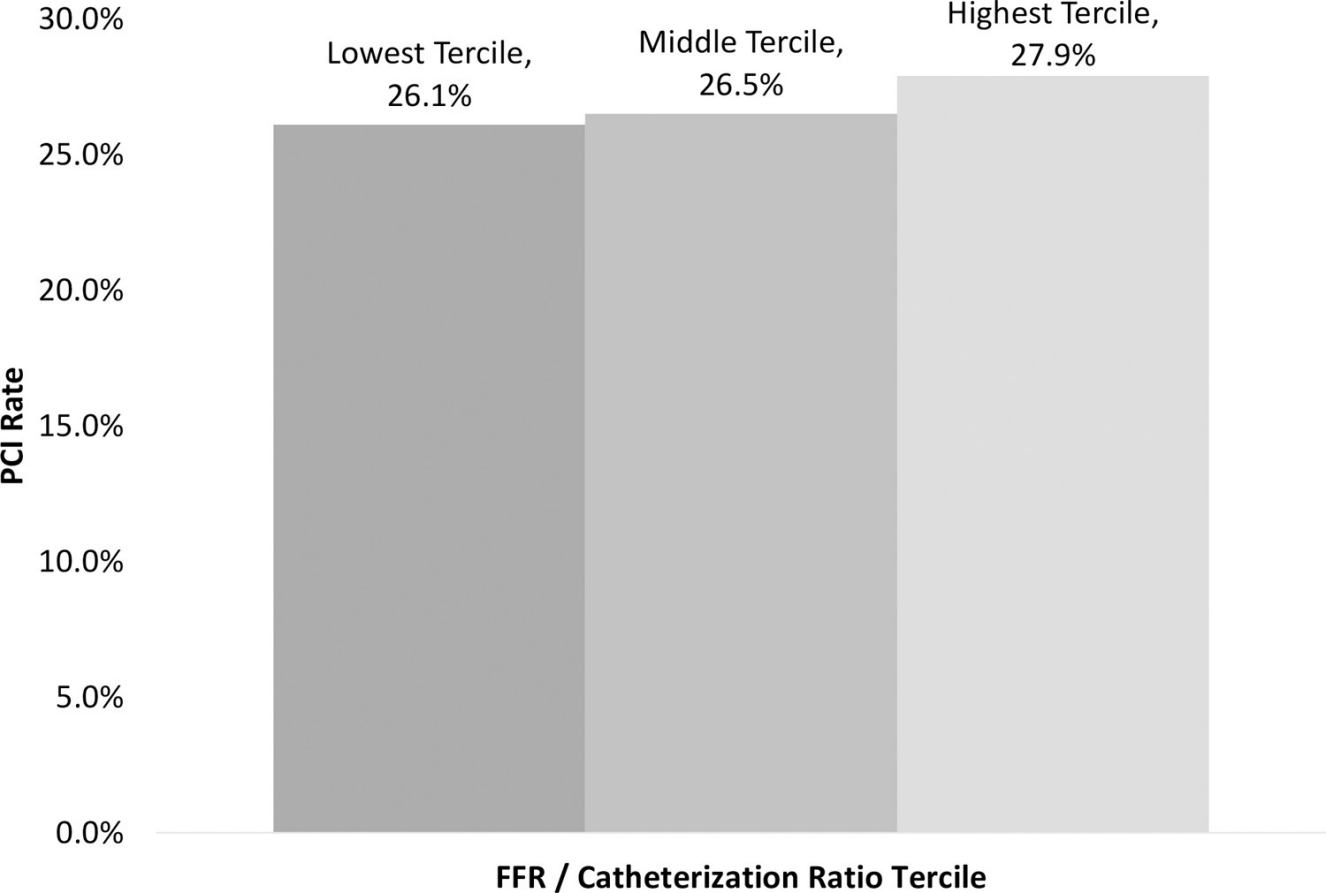
Relationship between a provider’s FFR / catheterization ratio tercile and PCI rate.

A Chi-Square test found a significant relationship between tercile and whether the catheterization was followed by a PCI (*P* = 0.02). The PCI rates were 26.1% (2,258/8,648), 26.5% (3,630/13,705), and 27.9% (2,666/9,567) for the lowest, middle, and highest terciles, respectively (Fig 2). A Chi-Square test did not find a significant relationship between tercile and whether the patient’s catheterization was followed by a CABG (*P* = 0.39).

Adjusted analysis from multivariate mixed effects logistic regression (Table 2) found there was a positive and significant association between downstream PCI and the catheterization provider’s FFR / catheterization ratio in the prior year (OR: 1.73; 95% CI: 1.26–2.37), as well as the mean patient age (OR: 1.78; 95% CI: 1.26–2.53) of the catheterization provider’s prior year patient cohort. There were likewise significant positive associations between several patient characteristics and downstream PCI: older age, male sex, and a history of more catheterizations in the prior twelve months.

**Table 2:** Adjusted results from multivariate mixed effects logistic regressions.

	PCI			CABG		
	*OR*	*95% CI*	*P value*	*OR*	*95% CI*	*P value*
***Provider Characteristics in Model***						
Cohort FFR / Catheterization Ratio in Prior Year	**1.73**	**1.26–2.37**	**< 0.01**	0.87	0.49–1.53	0.62
Place of Service (On-Campus Outpatient Hospital vs. Office)	1.16	0.92–1.47	0.21	0.97	0.65–1.45	0.89
Place of Service (Ambulatory Surgical Center vs. Office)	1.27	0.63–2.55	0.50	0.31	0.04–2.52	0.27
Cohort Mean Age in Prior Year	**1.78**	**1.26–2.53**	**< 0.01**	1.77	0.96–3.27	0.07
Cohort % Male in Prior Year	1.28	0.84–1.95	0.25	1.01	0.48–2.10	0.98
Cohort Local Median Income in Prior Year (% <$40,000)	1.06	0.83–1.34	0.64	0.87	0.58–1.32	0.51
Cohort Local Median Income in Prior Year (% >$80,000)	0.66	0.44–1.00	0.05	1.10	0.55–2.21	0.79
Cohort % Urban in Prior Year	1.11	0.93–1.32	0.24	0.93	0.69–1.26	0.66
***Patient Characteristics in Model***						
Age	**3.06**	**2.51–3.74**	**< 0.01**	**2.42**	**1.65–3.54**	**0.00**
Sex (Male)	**1.64**	**1.56–1.73**	**< 0.01**	**2.11**	**1.90–2.36**	**< 0.01**
# Catheterizations in Prior 12 Months	**20.41**	**10.42–39.97**	**< 0.01**	**0.02**	**0.00–0.12**	**0.00**
Local Median Income (<$40,000)	0.96	0.90–1.02	0.17	0.96	0.85–1.09	0.56
Local Median Income (>$80,000)	1.09	0.98–1.22	0.11	1.12	0.92–1.38	0.26
Urbanicity (Urban)	1.11	0.93–1.32	0.24	0.93	0.69–1.26	0.66
State Obesity Rate	1.41	0.38–5.28	0.61	7.01	0.70–70.14	0.10

Notes: Significant (P<0.05) values in bold. All continuous variables have been rescaled so that values fall between 0 and 1. Variables regarding panel percentages reflect the proportion of the panel with a characteristic.

Abbreviations: PCI: Percutaneous Coronary Intervention; CABG: Coronary Artery Bypass Graft; OR: Odds Ratio; CI: Confidence Interval; FFR: Fractional Flow Reserve.

Downstream CABG was positively associated with none of the provider characteristics. However, downstream CABG was significantly associated with several patient characteristics. CABG was positively associated with patient age and male sex, and negatively associated with the number of catheterizations received by the patient in the prior 12 months.

## Discussion

The findings of this study reinforce the notion that there are externally-observable provider characteristics that are statistically associated with patient outcomes. While health services researchers have examined geographic practice variation as a factor influencing variation in patient outcomes, this study spotlights the need for nongeographic provider variation to receive attention as well [19]. A study comparing the impact of patient preferences and physician beliefs on healthcare utilization in the contexts of cardiology and primary care found that the most important factor in determining treatment intensity was physicians’ beliefs about treatment intensity, as determined through a survey containing clinical vignettes [20]. Rather than using a survey, this study used an empirical method of procedural intensity (historical FFR utilization intensity, as evidenced by claims), and arrived at a congruent finding. Patients whose catheterization was performed by a procedurally-intensive provider, as evidenced by historical propensity to perform FFR, were significantly more likely to receive an intervention post-catheterization.

FFR provides physicians with additional information that can be used to better understand the hemodynamic significance of a stenosis, and potentially, to defer intervention. As its use is not indicated for all patients, this study examined the catheterization provider’s historic propensity to perform FFR, rather than if FFR was performed during the catheterization in question. While at the individual level, FFR may reduce the need for downstream intervention, at the population level, it is possible that physicians with the greatest propensity to perform FFR have a more intervention-oriented practice style which involves greater use of PCI, as in the adjusted model, an association was found between the FFR / catheterization ratio for the provider’s patient cohort in the prior year and the likelihood that a patient would receive PCI.

It is common for providers that perform catheterization to also perform PCI. PCI is sometimes performed by the provider while the patient is receiving catheterization (“ad hoc PCI”), and is sometimes performed at a later time (“staged PCI”). Since CABG is performed by a surgeon, and the provider performing catheterization is typically an interventional cardiologist and not a surgeon, the provider would likely need to make a referral for CABG to be performed. Thus, it is unsurprising that catheterization provider’s characteristics are associated with the performance of PCI but not CABG.

However, the findings of the study were unexpected, as existing literature suggests use of FFR can guide PCI decision making and enable PCI to be deferred [10, 13, 21]. It has been found that physicians may defer revascularization in response to findings from FFR assessment in at least 25% of cases [22]. As FFR findings can lead providers to both defer PCI and to perform it in contexts where PCI did not appear necessary through anatomical visualization, one potential explanation of the results of this study is that FFR findings led to the functional identification of stenoses. Furthermore, prior research has focused on patients that received FFR, not patients that merely were treated by providers with a propensity to perform it in the prior year.

In part due to the large sample size (31,920 patients), the analysis was powered to detect relatively small differences in patient demographics. Consequently, the descriptive statistics (Table 1) suggest that there was a significant association between providers’ propensity to perform FFR and all but one of the provider characteristics and patient characteristics examined. This suggests that providers with greater historic use of FFR treated a slightly different population of patients than providers with less historic use of FFR. Nonetheless, the demographic differences between the patients treated by the providers with the lowest and highest propensity to perform FFR were relatively small in magnitude, and demographic factors were incorporated as covariates in the multivariate regressions. It is possible that there is an unmeasured confounding factor that influenced both provider selection and post-catheterization outcomes.

The mean age of the patients in the catheterization providers’ catheterization patient cohort in the prior year was significantly and positively associated with whether a patient received PCI, even after controlling for the age of the patient being treated. Expectedly, the odds ratios from the patient age variables in the models were larger than the odds ratios from the provider’s prior year patient cohort age variables, suggesting that a patient’s own age has more of an impact on downstream care than the mean age of patients previously treated. While the age of the provider’s prior year patient cohort had a significant association with PCI, none of the other prior year patient cohort demographic factors–percentage male, percentage in ZIP codes with <$40,000 local median income, percentage in ZIP codes with >$80,000 local median income, and percentage urban–had a significant association with the outcomes examined.

In the regressions examining delivery of PCI, patients that were treated by a provider with an older prior year patient cohort were more likely to receive a PCI downstream, even after controlling for the patient’s own age. Prior research on preventive services found that patients belonging to older primary care panels were more likely to receive influenza vaccinations and HbA1c testing, suggesting that there may be a positive association between patient age and the aggressiveness of care [23]. Further research utilizing randomized designs is needed to determine whether providers with older patient cohorts have more procedurally-intensive styles of practice than providers with younger patient cohorts, after accounting for individual patient characteristics, as there may be endogeneity between patient cohort age and provider practice style.

### Limitations

The data analyzed pertain to patients with health plans from one organization, which constrained the demographics of the population evaluated by this study. Additionally, the data are not nationally representative, as they came from an organization whose health plan members predominantly live in the southern United States. Furthermore, the patient sample likely included only a portion of the providers’ patients; it is likely the providers included in the sample all treat numerous other patients that do not have a health plan from the organization which provided the data. As is the case with retrospective, claims-based research, the results may have been influenced by missing data, errors in coding, errors during claims processing, and factors that were not included in the models. Finally, this study was not able to assess whether the PCIs and CABGs that patients received (or did not receive) were clinically indicated.

## Conclusions

Healthcare providers’ historic procedural intensity and the demographics of their prior patients may impact the care that new patients receive. Further research is needed to examine whether these factors are associated with patients’ long-term healthcare utilization, morbidity, and mortality. If additional evidence is developed supporting their impact, these factors may be considered when selecting a provider.

## Supporting information

S1 Appendix Definitions. (DOCX)
