# Supplementary material for: The association between provider characteristics and post-catheterization interventions
Source: PLoS One. 2022 Apr 1;17(4):e0266544. doi: 10.1371/journal.pone.0266544 (PMC8975164; doi:10.1371/journal.pone.0266544)
Supplement: S1 Appendix — (DOCX) [file pone.0266544.s001.docx]

# **Appendix A: Definitions**

| **Diagnostic Catheterization** | | |
| --- | --- | --- |
| *CPT Code* | *Description* |  |
| 93452 | Left heart catheterization including intraprocedural injection(s) for left ventriculography, imaging supervision and interpretation, when performed |  |
| 93453 | Combined right and left heart catheterization including intraprocedural injection(s) for left ventriculography, imaging supervision and interpretation, when performed |  |
| 93454 | Catheter placement in coronary artery(s) for coronary angiography, including intraprocedural injection(s) for coronary angiography, imaging supervision and interpretation; |  |
| 93455 | Catheter placement in coronary artery(s) for coronary angiography, including intraprocedural injection(s) for coronary angiography, imaging supervision and interpretation; with catheter placement(s) in bypass graft(s) (internal mammary, free arterial venous grafts) including intraprocedural injection(s) for bypass graft angiography |  |
| 93456 | Catheter placement in coronary artery(s) for coronary angiography, including intraprocedural injection(s) for coronary angiography, imaging supervision and interpretation; with right heart catheterization |  |
| 93457 | Catheter placement in coronary artery(s) for coronary angiography, including intraprocedural injection(s) for coronary angiography, imaging supervision and interpretation; with catheter placement(s) in bypass graft(s) (internal mammary, free arterial, venous grafts) including intraprocedural injection(s) for bypass graft angiography and right heart catheterization |  |
| 93458 | Catheter placement in coronary artery(s) for coronary angiography, including intraprocedural injection(s) for coronary angiography, imaging supervision and interpretation; with left heart catheterization including intraprocedural injection(s) for left ventriculography, when performed |  |
| 93459 | Catheter placement in coronary artery(s) for coronary angiography, including intraprocedural injection(s) for coronary angiography, imaging supervision and interpretation; with left heart catheterization including intraprocedural injection(s) for left ventriculography, when performed, catheter placement(s) in bypass graft(s) (internal mammary, free arterial, venous grafts) with bypass graft angiography |  |
| 93460 | Catheter placement in coronary artery(s) for coronary angiography, including intraprocedural injection(s) for coronary angiography, imaging supervision and interpretation; with right and left heart catheterization including intraprocedural injection(s) for left ventriculography, when performed |  |
| 93461 | Catheter placement in coronary artery(s) for coronary angiography, including intraprocedural injection(s) for coronary angiography, imaging supervision and interpretation; with right and left heart catheterization including intraprocedural injection(s) for left ventriculography, when performed, catheter placement(s) in bypass graft(s) (internal mammary, free arterial, venous grafts) with bypass graft angiography |  |
| **FFR** |  |  |
| *CPT Code* | *Description* |  |
| 93571 | Intravascular Doppler velocity and/or pressure derived coronary flow reserve measurement (coronary vessel or graft) during coronary angiography including pharmacologically induced stress; initial vessel (List separately in addition to code for primary procedure) |  |
|  | |  |
| **CABG** |  |  |
| *CPT Code* | *Description* |  |
| 33510 | Coronary artery bypass, vein only; single coronary venous graft |  |
| 33511 | Coronary artery bypass, vein only; two coronary venous grafts |  |
| 33512 | Coronary artery bypass, vein only; three coronary venous grafts |  |
| 33513 | Coronary artery bypass, vein only; four coronary venous grafts |  |
|  |  |  |

| **PCI** |  |
| --- | --- |
| *CPT Code* | *Description* |
| 92920 | Percutaneous transluminal coronary angioplasty; single major coronary artery or branch |
| 92924 | Percutaneous transluminal coronary atherectomy, with coronary angioplasty when performed; single major coronary artery or branch |
| 92928 | Percutaneous transcatheter placement of intracoronary stent(s), with coronary angioplasty when performed; single major coronary artery or branch |
| 92933 | Percutaneous transluminal coronary atherectomy, with intracoronary stent, with coronary angioplasty when performed; single major coronary artery or branch |
| 92937 | Percutaneous transluminal revascularization of or through coronary artery bypass graft (internal mammary, free arterial, venous), any combination of intracoronary stent, atherectomy and angioplasty, including distal protection when performed; single vessel |
| 92941 | Percutaneous transluminal revascularization of acute total/subtotal occlusion during acute myocardial infarction, coronary artery or coronary artery bypass graft, any combination of intracoronary stent, atherectomy and angioplasty, including aspiration thrombectomy when performed, single vessel |
| 92943 | Percutaneous transluminal revascularization of chronic total occlusion, coronary artery, coronary artery branch, or coronary artery bypass graft, any combination of intracoronary stent, atherectomy and angioplasty; single vessel |
